# Uraemic brainstem encephalopathy mimicking ocular myasthenia: a case report

**DOI:** 10.1186/s12883-024-03626-y

**Published:** 2024-04-12

**Authors:** Pramith Ruwanpathirana, Thashi Chang

**Affiliations:** 1 Professorial Unit in Medicine, National Hospital of Sri Lanka, Colombo, 01000 Sri Lanka; 2 Department of Clinical Medicine, University of Colombo, 25, Kynsey Road, Colombo, 00800 Sri Lanka

**Keywords:** Renal failure, Dialysis, Ophthalmoplegia, Ptosis

## Abstract

**Background:**

Uraemia causes a generalised encephalopathy as its most common neurological complication. Isolated brainstem uraemic encephalopathy is rare. We report a case of fatigable ptosis and complex ophthalmoplegia in brainstem uraemic encephalopathy.

**Case presentation:**

A 22-year-old Sri Lankan man with end stage renal failure presented with acute onset diplopia and drooping of eyelids progressively worsening over one week. The patient had not complied with the prescribed renal replacement therapy which was planned to be initiated 5 months previously. On examination, his Glasgow coma scale score was 15/15, He had a fatigable asymmetrical bilateral ptosis. The ice-pack test was negative. There was a complex ophthalmoplegia with bilateral abduction failure and elevation failure of the right eye. The diplopia did not worsen with prolonged stare. The rest of the neurological examination was normal. Serum creatinine on admission was 21.81 mg/dl. The repetitive nerve stimulation did not show a decremental pattern. Magnetic resonance imaging (MRI) of the brain demonstrated diffuse midbrain and pontine oedema with T2 weighted/FLAIR hyperintensities. The patient was haemodialyzed on alternate days and his neurological deficits completely resolved by the end of the second week of dialysis. The follow up brain MRI done two weeks later demonstrated marked improvement of the brainstem oedema with residual T2 weighted/FLAIR hyperintensities in the midbrain.

**Conclusions:**

Uraemia may rarely cause an isolated brainstem encephalopathy mimicking ocular myasthenia, which resolves with correction of the uraemia.

**Supplementary Information:**

The online version contains supplementary material available at 10.1186/s12883-024-03626-y.

## Background

Neurological manifestations in uraemia are classified as central nervous system (CNS), peripheral nervous system (PNS) and myopathic manifestations [1]. CNS manifestations are the commonest and usually presents as generalised uraemic encephalopathy which is characterised by mental changes, disturbances of consciousness, seizures, asterixis, myoclonus, and electroencephalographic abnormalities [2]. PNS effects manifest as peripheral neuropathy and, myopathy as proximal muscle weakness. Although isolated brainstem encephalopathy with cranial nerve palsies and altered sensorium has been described before [3], uraemia causing ophthalmoplegia or ptosis with associated high signal intensities in the brainstem has not been previously reported. We describe a patient who presented with features mimicking ocular myasthenia due to isolated uraemic brainstem encephalopathy.

## Case presentation

A 22-year-old Sri Lankan man presented with acute onset diplopia, progressively worsening over one week. He had noted that his eyelids were drooping, especially towards end of the day. He did not experience dysphagia, dysarthria or limb weakness. He had been diagnosed with end stage renal failure (ESRF) due to obstructive uropathy one year ago. Although his serum creatinine had been 16.4 mg/dl (normal: 0.7 – 1.3) five months ago, he had not consented for renal replacement therapy.

**Fig. 1:**
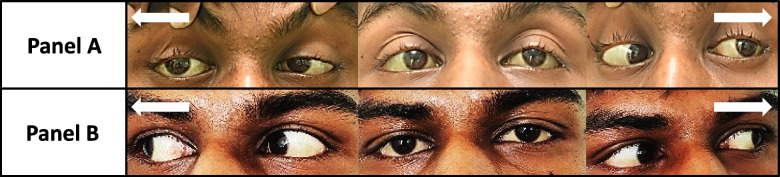
Eye movements before (**A**) and after (**B**) haemodialysis. **A** shows bilateral abduction failure (right medial rectus weaker than left) and the asymmetrical partial ptosis. The right eye is at a lower position compared to the left producing a skew deviation. **B** shows complete resolution of the ocular signs. White arrows indicate the direction the patient is looking at

On examination, he had a fatigable bilateral asymmetrical partial ptosis (additional file 1) and a positive curtain sign (manually raising the more ptotic lid causes increased ptosis on the opposite side), but the Cogan’s lid twitch and peek signs were negative. There was a complex external ophthalmoplegia without nystagmus (Fig. 1 – Panel A). The pupils were normal. Limb power was 5/5 (modified medical research council score) in all four limbs, no cerebellar signs were noted and the sensation to pin prick and joint position sense was intact. The rest of the examination were normal apart from severe pallor. The blood pressure was 130/80 mmHg. The bedside ice-pack test for ocular myasthenia gravis was negative.

The biochemical parameters on admission are given in the Table 1.

**Table 1:** Biochemical investigations on admission

Parameter	Value	Normal Range
Creatinine	20.81 mg/dl	0.7—1.3
Na +	133 mmol/l	135—145
K +	4.4 mmol/l	3.6—5.2
ESR	32 mm/1st hour	0 – 22
CRP	2 mg/l	< 6
Albumin corrected Ca^2+^	6.2 mg/dl	8.5—10.2
pH	7.12	7.35 – 7.45
pCO2	7.7 mmHg	35—45
HCO_3_^−^	2.5 mmol/l	22 – 26
Base Excess	-27.1 mmol/l	(-2) – (+ 2)

After correcting the hypocalcaemia, haemodialysis was commenced because of the severe acidosis and continued on an alternate day frequency.

In the evaluation for the cause of the patient’s complex ophthalmoplegia and fatigable ptosis, the following investigations were done (Table 2).

**Table 2:** Investigations for complex ophthalmoplegia and fatigable ptosis

Investigation	Result	Comment
Repetitive nerve stimulation (right ulnar nerve, left facial nerve and left spinal accessory nerve)	No decremental pattern demonstrated	See Supplementary material 1 for the detailed report
MRI brain	T2W/FLAIR high signal intensities involving the internal capsule, midbrain, pons, upper medulla and middle cerebellar peduncles No diffusion restriction.	Figure 2 – Panel A
Cerebrospinal fluid (CSF)	Protein – 85 mg/dl Glucose – 52 mg/dl (plasma glucose – 94 mg/dl) Cells – none	A non-reactive CSF

Acetylcholine receptor antibodies were not tested due to resource constraints.

**Fig. 2:**
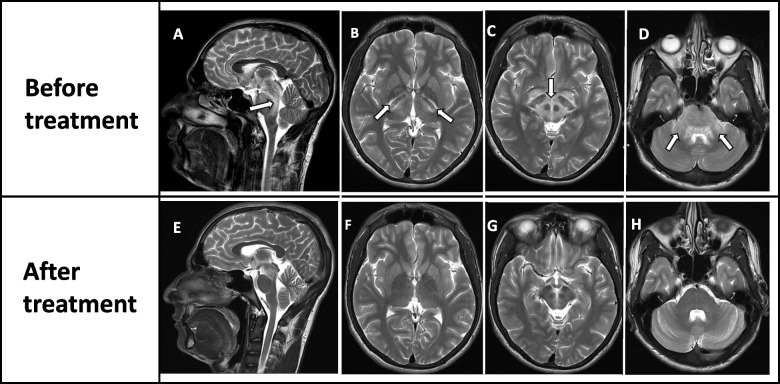
T2W MRI images of the brain before and after haemodialysis. The hyperintensities seen in the mid brain and pons (sagittal) (**A**), internal capsule (**B**), midbrain (except substantia nigra, red nucleus, and superior colliculus) (**C**) and middle cerebellar peduncles (**D**) are markedly reduced after haemodialysis (**E** to **H**)

The patient’s ophthalmoplegia improved completely by the end of the first week after initiating haemodialysis and the ptosis resolved by the end of the second week (Fig. 1 – Panel B). The MRI of the brain repeated after two weeks demonstrated marked improvement in the previously noted brainstem abnormalities with residual mild T2W/FLAIR hyperintensities in the midbrain. T2W hyperintensities in the internal capsule and cerebellar peduncles had completely resolved (Fig. 2 – Panel B).

## Discussion

This is the first report of uraemia causing a predominant brainstem encephalopathy manifesting as external ophthalmoplegia and fatigable ptosis; mimicking ocular myasthenia. Although a case of bilateral external ophthalmoplegia associated with uraemia which resolved with dialysis was described in 1994 [4], brainstem hyperintensities were not demonstrated due to absence of MRI facilities at that point of time. The neurological manifestations of our patient were attributed to uraemia on the basis that ptosis and ophthalmoplegia completely resolved paralleled by improvement on MRI with the correction of the uraemia following regular haemodialysis. The absence of a reactive CSF ruled out an inflammatory aetiology of the brainstem pathology accounting for the patient’s clnico-radiological manifestations. Apart for fatigability and enhanced ptosis, the patient did not have any other feature to suggest autoimmune myasthenia gravis. Furthermore, brainstem MRI abnormalities and a rapid resolution with haemodialysis are not consistent with myasthenia gravis. Posterior reversible encephalopathy syndrome was considered unlikely as the blood pressure was normal, the occipital lobes were spared and the ADC map was normal. Wernicke’s encephalopathy, although common among patients with CKD was considered unlikely as ataxia and encephalopathy was absent and the symptoms improved without thiamine replacement.

The exact mechanisms and the molecules responsible for the neurological manifestations of uraemia have not been identified. It is postulated that there are over 130 neurotoxic compounds, that accumulate in the body in renal failure [5]. Uric acid, indoxyl sulphate, p-cresyl sulphate, interleukin 1-β, interleukin 6, TNF-α, and parathyroid hormone are a few compounds implicated in the cerebro-renal interactions [6]. Guanidine compounds are considered as potential neurotoxins [7]. Amino acid metabolites that accumulate in renal failure cross the blood brain barrier and interfere with neuro-transmitter handling in CNS [8]. Furthermore, metabolic acidosis and electrolyte imbalances interfere with normal CNS functions [8].

Bilateral asymmetrical ptosis due to a midbrain pathology has been previously described [9, 10]. This has been attributed to a single nuclear complex (centro.-caudal subnucleus of the oculomotor nuclear complex) that innervates the levator palpebrae superioris muscles bilaterally [11] However, the cause of fatigability of the ptosis, a hallmark sign of myasthenia gravis, observed in our patient remains elusive given that his lesion was central.

Despite the brainstem encephalopathy, our patient did not manifest long tract signs or reduced level of consciousness. This may be explained on the assumption that the high signal intensities in T2 weighted images were due to oedema and not due to inflammation. We hypothesise that the oculomotor nucleus may be excessively susceptible to oedema or uraemia or both in combination.

Neuro-imaging manifestations of uraemic encephalopathy are diverse. Increased signal intensities on T2WI, FLAIR in the region of the basal ganglia and the ‘Lentiform fork sign’ (hyperintense lines in the shape of a fork in the lateral and medial boundaries of the putamen and in-between the globus pallidum externa and interna on T2W sequence), has been described in uraemic encephalopathy [12]. These features were not found in our patient. Although isolated brainstem hyperintensities in uraemia as seen in our patient has been reported once previously in a Chinese patient who presented with unconsciousness, anisocoria and hyperreflexia [3], manifestation with ptosis and ophthalmoplegia has not been previously described.

## Conclusion

In conclusion, our case report adds to the varied spectrum of neurological manifestations reported in uraemia and emphasises the importance to test renal functions in patients presenting with features suggestive of ocular myasthenia.

### Supplementary Information

**Additional file 1. **Fatigability of ptosis. When the patient is asked to look up, increasing of ptosis can be seen. The forehead is wrinkled, which indicates frontalis overactivity to compensate for the fatigable ptosis.

**Additional file 2. **Repetitive Nerve Stimulation Test Report. The repetitive nerve stimulation test doesn’t demonstrate a decremental pattern.

## Data Availability

Not applicable.

## Abbreviations

CNS: Central nervous system
CSF: Cerebrospinal fluid
CT: Computed tomography
FALIR: Fluid attenuated inversion recovery
PNS: Peripheral nervous system
T2W: T2 weighted
