# Supplementary material for: Uraemic brainstem encephalopathy mimicking ocular myasthenia: a case report
Source: BMC Neurol. 2024 Apr 12;24:121. doi: 10.1186/s12883-024-03626-y (PMC11010437; doi:10.1186/s12883-024-03626-y)
Supplement: Supplementary file 2 — Additional file 2. Repetitive Nerve Stimulation Test Report. The repetitive nerve stimulation test doesn’t demonstrate a decremental pattern. [file 12883_2024_3626_MOESM2_ESM.pdf]

Supplementary material 2

Results of repetitive nerve stimulation

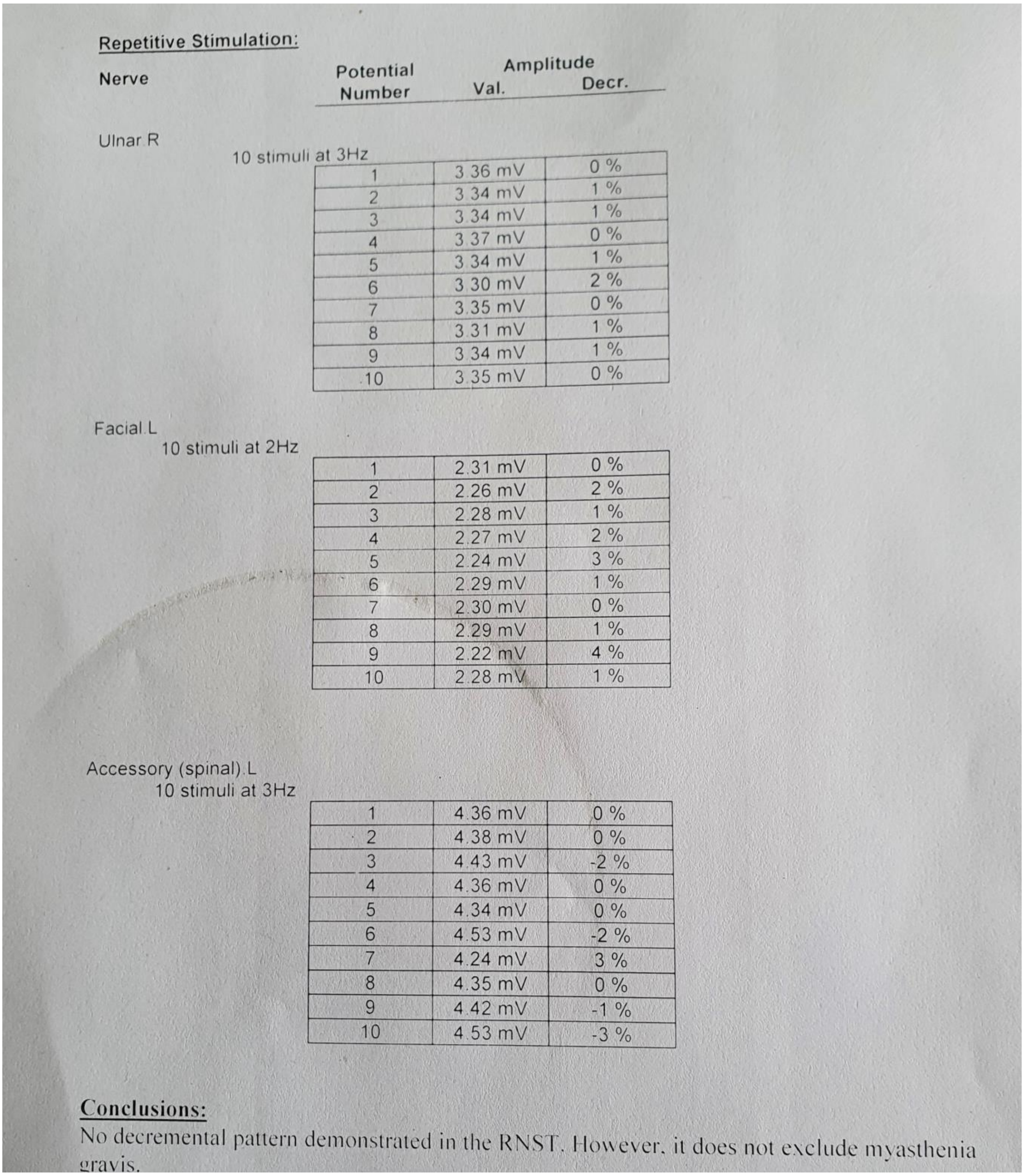

Repetitive Stimulation:

Nerve	Potential Number	Amplitude Val.	Amplitude Decr.
Ulnar R			
10 stimuli at 3Hz	1	3.36 mV	0 %
	2	3.34 mV	1 %
	3	3.34 mV	1 %
	4	3.37 mV	0 %
	5	3.34 mV	1 %
	6	3.30 mV	2 %
	7	3.35 mV	0 %
	8	3.31 mV	1 %
	9	3.34 mV	1 %
	10	3.35 mV	0 %
Facial L			
10 stimuli at 2Hz	1	2.31 mV	0 %
	2	2.26 mV	2 %
	3	2.28 mV	1 %
	4	2.27 mV	2 %
	5	2.24 mV	3 %
	6	2.29 mV	1 %
	7	2.30 mV	0 %
	8	2.29 mV	1 %
	9	2.22 mV	4 %
	10	2.28 mV	1 %
Accessory (spinal) L			
10 stimuli at 3Hz	1	4.36 mV	0 %
	2	4.38 mV	0 %
	3	4.43 mV	-2 %
	4	4.36 mV	0 %
	5	4.34 mV	0 %
	6	4.53 mV	-2 %
	7	4.24 mV	3 %
	8	4.35 mV	0 %
	9	4.42 mV	-1 %
	10	4.53 mV	-3 %

Conclusions:

No decremental pattern demonstrated in the RNST. However, it does not exclude myasthenia gravis.

Repetitive nerve stimulation was done in the right ulnar, left facial and left spinal accessory nerves. A decremental pattern was not observed.
